# Supplementary material for: Diversification of Pseudomonas aeruginosa Biofilm Populations under Repeated Phage Exposures Decreases the Efficacy of the Treatment
Source: Microorganisms. 2024 Sep 12;12(9):1880. doi: 10.3390/microorganisms12091880 (PMC11434582; doi:10.3390/microorganisms12091880)
Supplement: Supplementary file 1 [file microorganisms-12-01880-s001.zip › Supplementary Material_revised.pdf]

Supplementary Material

Diversification of *Pseudomonas aeruginosa* Biofilm Populations under Repeated Phage Exposures Decreases the Efficacy of the Treatment

Mark Grevsen Martinet [1], Mara Lohde [1], Doaa Higazy [2], Christian Brandt [1,3], Mathias W. Pletz [1,3], Mathias Middelboe [4,5], Oliwia Makarewicz [1,3,†] and Oana Ciofu [2,*,†]

[1] Institute of Infectious Diseases and Infection Control, Jena University Hospital, 07747 Jena, Germany; mark.martinet@med.uni-jena.de (M.G.M.); mara.lohde@med.uni-jena.de (M.L.); christian.brandt@med.uni-jena.de (C.B.); mathias.pletz@med.uni-jena.de (M.W.P.); oliwia.makarewicz@med.uni-jena.de (O.M.)
[2] Costerton Biofilm Center, Department of Immunology and Microbiology, Faculty of Health and Medical Sciences, University of Copenhagen, 2200 Copenhagen, Denmark; doaa.mohamed@agr.cu.edu.eg
[3] Leibniz Center for Photonics in Infection Research (LPI), 07745 Jena, Germany
[4] Marine Biological Section, Department of Biology, University of Copenhagen, 3000 Helsingør, Denmark; mmiddelboe@bio.ku.dk
[5] Department of Biology, University of Southern Denmark, 5230 Odense, Denmark
* Correspondence: ociofu@sund.ku.dk
† These authors contributed equally to this work as senior authors.

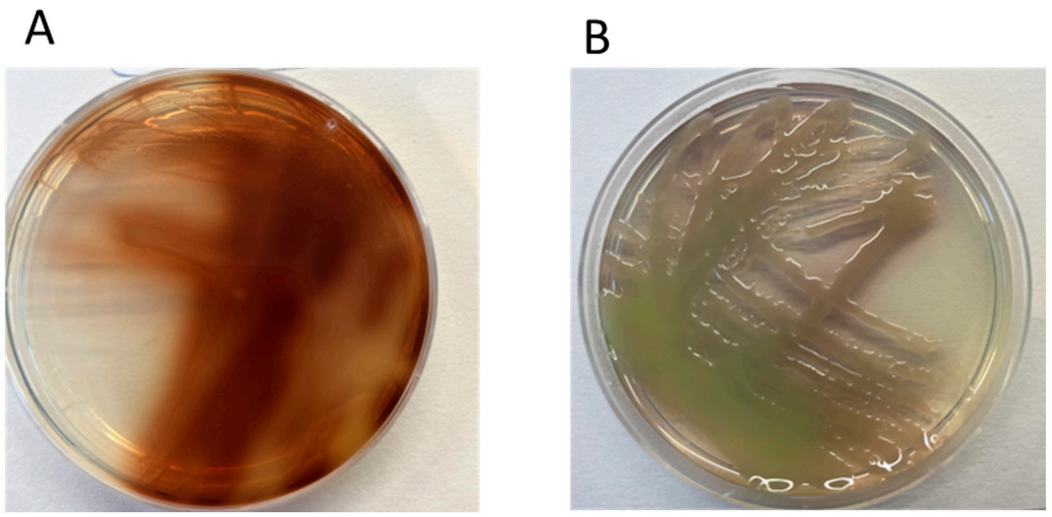

Figure S1. Representative pictures of *P. aeruginosa* culture producing pyomelanin (A) and alginate (mucoid colony) after bacteriophage treatment of biofilm grown on LB agar plates

Supplementary Results

Additional genetic alterations in clones collected from biofilm exposed to phages

Upon comparing phage-treated clones with their ancestors, additional alterations were discerned that do not exhibit a direct correlation with the phage susceptibility phenotype (Figure 5 A to E). The predominant mutation identified in five resistant and three sensitive clones of the clinical isolate CF341_08 was the in-frame insertion Leu486insGluPro in the *dnaX* gene encoding for the DNA polymerase III subunits γ and τ. The DNA polymerase III is the major conserved DNA replication holoenzyme in prokaryotes. The mutation results in the extension of the GluPro compositionally biased (CB) region, consisting of 13 GluPro repeats, by one additional repeat. The consequences of this alteration remain uncertain, although it appears more probable that it represents a common mutation within this gene, given its occurrence in a highly repetitive sequence.

In one NP3-resistant clone of PA14, a synonymous mutation manifested in the *ubiA* gene encoding for 4-hydroxybenzoate octaprenyltransferase, the second enzyme in the pathway of ubiquinone biosynthesis. This mutation does not impart any consequential alterations to the protein's functionality, but potential impacts related to codon usage leading to a diminished ubiquinone content cannot be entirely ruled out. In general, these mutations surfaced early in the biofilm population (after 24 h), and were unique to each strain lineage, underscoring their spontaneous nature.

The majority of PAO1 subjected to NP3 and M32 treatment exhibited a Ser125Gly substitution in RluA and a frameshift at Thr46 in Wzy, independently of treatment duration or phage susceptibility phenotype. The RluA modifies primary the U-nucleotides in the tRNA [26]. The Ser125Gly substitution in RluA occurred between two β-sheets. A *blastp* analysis revealed the presence of Gly at this position in RluA proteins from other *P. aeruginosa* strains, suggesting that this alteration is likely inconsequential to the protein's function.

Various frameshifts or nonsense mutations, all resulting in the loss of function, were found in PilY1 in both in M32-sensitive and M32-resistant clones, as well as in one NP3-resistant clone of PAO1. PilY1 is a component of the inner membrane sub-complex of the type IV pilus assembly machinery [16]. As PilY1 is situated internally, it is improbable that it plays a role in phage recognition, as it is not exposed to the external environment. Malfunctions in this protein

do not seem to induce alterations in other recognition patterns associated with pili that typically accompany phage resistance.

The predominant mutation observed early in the biofilm population of CF341_06, present in both phage-sensitive and -resistant clones, involved a duplication of Pro at amino acid position 289 in the type VI secretion system (T6SS) forkhead-associated protein Fha1. This duplication occurred in the C-terminal end of the distorted loop, not in the forkhead-associated domain. Fha1 is located in the inner membrane at the T6SS-membrane core complex and activates pilus assembly upon phosphorylation, triggered by perturbations of the outer membrane [25]. Given that the Pro duplication occurs at the termination of the GlnPro compositional bias region (consisting of 11 GlnPro repeats), a malfunction of Fha1 and subsequent absence of T6SS pili is deemed rather unlikely, although it cannot be completely excluded. Importantly, Fha1 is not accessible to the phage as a receptor, making it also improbable that this alteration impacts host recognition by the NP3 phage.

Table S2. Information on CF clinical isolates (CF 341)

Patient CF 341		
Isolate	CF341_06_NM	CF341_08_NM
Year of isolation	2006	2008
Nonmucoid (N) phenotype	NM	NM
Infection status at time of isolation	Intermittently colonised	Chronically infected
P. aeruginosa precipitin	1	28
Weeks of peroral/inhalation/ intravenous antibiotic therapy	0	Po: 6<br>IV: 14
Sensitivity towards ciprofloxacin of the bacterial isolates in planktonic form (µg/mL) determined by e-test	0.064	0.380
Generation time *in vitro* (min.)	42.2	47

Table S3. Host-range NP3 determined by spot assay on environmental, reference and CF clinical isolates.

Source	*P. aeruginosa* strain	Lysis by NP3
environmental	PS F8	+
environmental	PS F10	+
environmental	PS 119X	+
environmental	PS M4	+
reference	PAO1	+
reference	PDO300 (M)	+
reference	PA14	+
clinical	CF 46 476a/88	+
clinical	CF 46 476b/88	+
clinical	CF46 52329a/97	+
clinical	CF 46 52329b/97	+
clinical	CF46 1395a/03	+
clinical	CF 46 1395b/03	-
clinical	CF 30 19731a/92	+
clinical	CF30 19731b/92	+
clinical	CF30 75887a/01	+
clinical	CF30 75887b/01	+
clinical	CF128 1398a/92	+
clinical	CF128 1398b/92	+
clinical	CF128 8503a/02	+
clinical	CF128 8503b/02	0
clinical	CF 89 15061/78	+
clinical	CF238 82847b/01	+
clinical	CF 341_ 28898_06	+
clinical	CF 341_43885A 08A (M)	(+m)
clinical	CF 341 43885B_08 (NM)	+
clinical	CF 405_56281A_05	+
clinical	CF 405_3299_10	+
clinical	CF 408_19970_06	+
clinical	CF 408_38574A_06	-
clinical	CF 414_15321_06	+
clinical	CF 414_18275_08	+

clinical	CF 414_36687_09	-
clinical	CF 431_24293_08	+
clinical	CF 431_7494_11	+
clinical	CF 483_7161A_04	+
clinical	CF 483_2728A_06	(+)
clinical	CF 483_2728B_06	(+)
clinical	CF 519_19637B_07	+
clinical	CF 519_17006_1_11	+
clinical	CF 519_17006_2_11	+
clinical	CF 544_55286B_10	+
clinical	CF 544_60163B_11	+
clinical	CF 544_60163A_11	+
clinical	CF 312_16704_91	+
clinical	CF 312_20441A_95	+
clinical	CF 312_20441B_95	+

Information on the method: For the spot test, a fresh bacteria culture was adjusted to an OD600 of 0.1 (approximately 10^9 CFU/mL) and 0.3 mL of the bacteria suspension was mixed with 4 mL of top agar and poured onto an LB plate. After solidification, 10 µL of 10-fold serial diluted phage stock was spotted onto the plate and incubated overnight. The presence of plaques/halos indicated the susceptibility
